# Impact of Elevated Plasma Serotonin on Global Gene Expression of Murine Megakaryocytes

**DOI:** 10.1371/journal.pone.0072580

**Published:** 2013-08-27

**Authors:** Charles P. Mercado, Stephanie Byrum, Marjorie L. Beggs, Endrit Ziu, Preeti Singh, Vinay R. Raj, Randy S. Haun, Fusun Kilic

**Affiliations:** 1 Biochemistry and Molecular Biology, College of Medicine, University of Arkansas for Medical Sciences, Little Rock, Arkansas, United States of America; 2 Medical Genetics, University of Arkansas for Medical Sciences, Little Rock, Arkansas, United States of America; 3 Pharmacogenomics Core, College of Medicine, University of Arkansas for Medical Sciences, Little Rock, Arkansas, United States of America; 4 Pharmaceutical Sciences, College of Pharmacy, University of Arkansas for Medical Sciences, Little Rock, Arkansas, United States of America; Goethe University Frankfurt, Germany

## Abstract

**Background:**

Serotonin (5-HT) is a biogenic amine that also acts as a mitogen and a developmental signal early in rodent embryogenesis. Genetic and pharmacological disruption of 5-HT signaling causes various diseases and disorders via mediating central nervous system, cardiovascular system, and serious abnormalities on a growing embryo. Today, neither the effective modulators on 5-HT signaling pathways nor the genes affected by 5-HT signal are well known yet.

**Methodology/Principal Findings:**

In an attempt to identify the genes altered by 5-HT signaling pathways, we analyzed the global gene expression via the Illumina array platform using the mouse WG-6 v2.0 Expression BeadChip containing 45,281 probe sets representing 30,854 genes in megakaryocytes isolated from mice infused with 5-HT or saline. We identified 723 differentially expressed genes of which 706 were induced and 17 were repressed by elevated plasma 5-HT.

**Conclusions/Significance:**

Hierarchical gene clustering analysis was utilized to represent relations between groups and clusters. Using gene ontology mining tools and canonical pathway analyses, we identified multiple biological pathways that are regulated by 5-HT: (i) cytoskeletal remodeling, (ii) G-protein signaling, (iii) vesicular transport, and (iv) apoptosis and survival. Our data encompass the first extensive genome-wide based profiling in the progenitors of platelets in response to 5-HT elevation *in vivo*.

## Introduction

Serotonin (5-HT, 5-hydroxytryptamine) is a multifunctional signaling molecule that acts differently in different organ-systems, in a concentration-dependent manner. 5-HT plays roles, not only in adult life, but in development as well [1]–[8]. Recent evidence reveals that 5-HT is a growth factor, promoting cellular proliferation and neurogenesis in the primordial enteric nervous system [4]. In mature animals, 5-HT acts in various ways as a paracrine signaling molecule and as an enteric neurotransmitter that initiates peristaltic and secretory reflexes and also acts as a hormone released from the small intestine that regulates bone formation and exerts effects on glucose and lipid metabolism [5]. 5-HT interacts with innate and acquired immunoeffector cells to promote defensive or pathophysiological inflammation and within the enteric nervous system 5-HT is neuroprotective and enhances neurogenesis, effectively protecting neurons from the detrimental effects of inflammation [3]. Furthermore, 5-HT acts on the liver to enhance regeneration, and also promotes fibrosis and fat deposition [2]. During early embryogenesis 5-HT is a mitogen and plays a crucial modulator role in the function and dysfunction of the serotonergic system [6]–[9] via modulating cell division, neuronal migration, cell differentiation, and synaptogenesis through activating 5-HT receptors and downstream signal transduction pathways [6]–[9]. Maternal 5-HT is important in fetal development and 5-HT plays important roles in vascular remodeling and maintenance in the cardiovascular (CV) system.

Today, clinical studies agree that plasma 5-HT levels become elevated in a variety of pathologies including hypertension, coronary artery disease, valvular heart disease, atherothrombosis, and myocardial infarction. Reports of blood 5-HT concentrations in patients with CV diseases indicate 5-HT rises of less than two to four-fold in most chronic disorders including hypertension, diabetes, and coronary artery disease [10]–[14]. More profound 5-HT elevations averaging 10 to 16-fold higher than normal individuals have been detected during surgeries for acute coronary conditions [11], [12].

Studies provided evidence for the involvement of elevated plasma 5-HT in platelet biology at the protein level. Through binding to small GTPases or G-coupled 5-HT_2A_ receptor subtypes, 5-HT is associated in membrane trafficking of several proteins in platelets and other cells via receptor (5-HT_2A_)-dependent [15]–[18] or -independent signaling pathways [19]–[22]. 5-HT activates downstream signaling pathways that have diverse functional consequences–cytoskeletal remodeling, formation of lamellipodia and filopodia, and shape change, which are hallmarks of platelet activation [21]. 5-HT_2A_-dependent release of α-granules during platelet activation is one example for a 5-HT_2A_-dependent indirect effect of 5-HT on platelet biology [16].

Platelets are derived and released from the progenitor megakaryocyte (MK) in the bone marrow, the downstream effects of the changes in the MK genes expression levels can be directly observed at the platelet level. Although at the protein level the different roles of 5-HT have been explored and reported, neither its involvement in gene expression nor the identifications of genes which are affected by high levels of plasma 5-HT have been explored yet.

In an attempt to identify the genes altered by 5-HT signaling pathways, we utilized the mRNA derived from MKs of mice infused with 5-HT or saline. MKs are the direct progenitors of platelets in the peripheral circulation. Using microarray technology we performed genome-wide based profiling of mature and immature MKs derived from 5-HT- and saline-infused mice. In particular, bone marrow CD41-derived MKs were isolated and purified from mice infused with saline or 5-HT for 24 hours. The pattern of gene expression in both groups was compared and differentially expressed genes were identified. Following gene ontology mining and pathway analyses, we have identified novel genes and several signaling pathways that are altered by elevated levels of plasma 5-HT. Since platelets are derived and released from the progenitor MK in the bone marrow, the downstream effects of changes in the expression levels of MK genes can be observed directly on platelets. In this communication, the implications of platelet function as a predictor of changes in MK gene expression are discussed.

## Materials and Methods

### Animals and Treatments

Animal procedures were approved by the University of Arkansas for Medical Sciences Animal Care and Use Committee. Adult C57BL/6J male mice (Harlan, Indianapolis, IN), 10–12 weeks old with body weight between 25–30 grams were used in the present study. Animals were maintained in rooms at 24°C, with 40% humidity and a 12-h light/dark cycle and were provided food and water *ad libitum* until used. Osmotic pumps were implanted subcutaneously between the scapulae of mice anesthetized using isofluorane (2.5% at 1.5 L/min oxygen) [18]. Animals in the treatment group were infused with 25.8 mM 5-HT (Sigma, St. Louis, MO) using Alzet minipumps (model 1003D, Cupertino, CA) at a rate of 1.66 µg/kg/hr over 24 hours. This treatment elevates the plasma 5-HT level as verified by ELISA (Labor Diagnostika Nord, Nordhorn, Germany) [10], [18]. Mice in the control group were infused with saline over 24 hours.

### Megakaryocyte Isolation

From anesthetized mice, the femurs were dissected and marrow cells were collected in MK buffer (Ca^2+^, Mg^2+^-free phosphate-buffered saline [PBS] containing 3% bovine serum albumin [BSA], 5.5 mM D-glucose, 10.2 mM trisodium citrate, and 10 µM prostaglandin E1 [PGE1] and filtered with 90 µm nylon membranes (Fisher Scientific, Pittsburgh, PA). Bone marrow cells were pooled (n = 3–4 mice per sample) and enriched using Percoll (Sigma, St. Louis, MO) gradients. After washing, the cells were incubated with a monoclonal anti-mouse antibody directed against platelet glycoprotein IIb (CD41^+^; BD Biosciences, San Jose, CA) and suspended with immunomagnetic beads (Dynabeads, Invitrogen Corp., Carlsbad, CA) coated with sheep anti-mouse IgG antibody [23], [24]. The CD41^+^ antibody has high specificity for mature and immature MKs [25], [26] and this isolation technique yields at least 96.6% purity of MKs [27].

### RNA Processing

Immunomagnetic beads bound to MK cells were suspended in RLT lysis buffer (Qiagen Inc., Valencia, CA) and sonicated briefly. The beads were separated from the supernatant using a magnet and total RNA was isolated using an RNA isolation kit as per the manufacturer’s protocol (Qiagen Inc., Valencia, CA). Purified RNA was checked for integrity and quantified using a Pico Total RNA Bioanalyzer kit (Agilent RNA 6000 Pico Kit) and Agilent 2100 BioAnalyzer (Agilent Technologies Inc., Santa Clara, CA).

### Microarray Analysis

Biotin-cRNA was prepared from high quality total RNA samples with a TargetAmp Nano-g Biotin-aRNA labeling kit for the Illumina System (Epicentre Biotechnologies, Madison, WI). Subsequently, cRNA was hybridized to the array for 16 hours. After several wash steps, the array chip was stained with streptavidin-Cy3 and scanned using an Illumina BeadArray reader (Illumina, San Diego, CA).

### Bioinformatics

Data were analyzed using the gene expression module of Illumina Genome Studio software to assess quality of RNA and hybridization. The raw gene expression data were generated after quality control analysis. The expression intensities were median-normalized after log transformation. Genes based on a mean expression value cut-off across all samples were selected for differential gene expression analysis. Fold change (FC) above 1.5 (with p-value <0.05) was defined as differentially expressed between the two populations. The fold change in transcription expression levels were expressed as the log_2_ ratio [signal log ratio (SLR); a log_2_ ratio of 1 is equal to FC of 2]. In order to categorize the differential gene expression, hierarchical clustering was performed with average linkage and Euclidean distance metrics. All analyses were conducted using the R statistical environment [28]. Gene ontology mining and pathway analyses were carried out using MetaCore software (GeneGo, Inc., St. Joseph, MI). The microarray data have been submitted to the GEO repository (GSE37273).

### Platelet preparation

Blood was extracted by cardiac puncture from anesthetized mice using a syringe with a 19-gauge needle in the presence of the anticoagulant sodium citrate (3.8% v/v). Platelets were buffered with Ca^2+^-free Tyrode-HEPES (134 mM NaCl, 0.34 mM Na_2_HPO_4_, 2.9 mM KCl, 12 mM NaHCO_3_, 20 mM HEPES, 5 mM glucose, 1 mM MgCl_2_, pH 7.3) at a ratio of 2:1. Blood was centrifuged at 200 *g*, 20°C for 10 min to pellet the platelets. Platelets were counted using a Hemavet 950 (Drew Scientific, Waterbury, CT) and normalized accordingly [10], [18]. Where necessary, platelets were separated from the blood plasma via centrifugation at 1000 *g* for 10 min and resuspended in Ca^2+^-free Tyrode-HEPES or PBS. 5-HT infusion was confirmed by quantifying the amount of 5-HT in platelets and plasma by competitive ELISA techniques [18].

### FACS analysis

For flow cytometric analysis, 2.5×10^7^ platelets were processed for each staining at room temperature. Cells were blocked with PBS containing 1% BSA for 30 min before being incubated with primary antibodies for 30 min, followed by corresponding secondary antibodies for 30 min. Either secondary antibody alone or Alexa Fluor/FITC-conjugated isotype matched antibodies were used as controls. The tubes were rotated in the dark for 30 min. Four hundred microliters of 1% formaldehyde was added to stop the reaction and the samples were analyzed using a flow cytometer (FACS Calibur, Becton-Dickson, San Jose, CA) at the University of Arkansas for Medical Sciences Microbiology and Immunology Flow Cytometry Core Laboratory [18]. Fifty thousand events gated based on their forward and side scatter profiles were analyzed. Mean fluorescence intensity (MFI) was also recorded. Flowjo 7.2.5 software (Tree Star, Inc., Ashland, OR) was used to analyze the results. FACS analysis was performed at least 3 times for each marker [18].

### 5-HT secretion of permeabilized platelets

Washed platelets isolated from saline- and 5-HT-treated mice were permeabilized following a modified protocol previously described by Shirakawa *et al.* [16]. Briefly, platelets were resuspended in EGTA buffer (145 mM NaCl, 5 mM KCl, 1 mM MgCl_2_, 10 mM HEPES, pH 7.4, 10 mM glucose, 2 mM EGTA) then incubated with 2 nM 5-HT/PBSCM (PBS plus 0.1 mM Ca and 0.1 mM Mg) for 30 min. Platelets were pelleted by centrifugation and resuspended in pre-warmed buffer A (4 mg/mL BSA, 5 mM ATP, 8 mM creatine phosphate, 50 µg/mL creatine phosphokinase) and immediately transferred to a 30°C thermo block. Streptolysin-O (SLO, 0.6 µg/ml) was added to form pores in the plasma membrane. Stimulation buffer (50 mM HEPES/KOH pH 7.4, 78 mM KCl, 4 mM MgCl_2_, 2 mM EGTA, 20 mM CaCl_2_) was added to the samples followed by addition of stop buffer (ice-cold buffer A). 5-HT in the supernatant was collected by centrifugation at 5000 *g* for 5 min. One mL of *o*-Phaldialdehyde (Sigma St. Louis P1378 0.5% W/V) was prepared and added to 250 mL of supernatant and allowed to react with 5-HT for 10 min at 95°C. After centrifugation and several wash steps with chloroform, samples were excited at 355 nm and emission at wavelength 475 nm was recorded [29]. Statistical analysis was conducted using Student’s *t*-test.

### Data analysis

Nonlinear regression fits of experimental and calculated data were performed using Origin software, which uses the Marquardt-Levenberg non-linear least squares curve fitting algorithm. Each figure shows a representative experiment that was performed at least three times. The statistical analyses given in the Results section is from multiple experiments. Data with error bars are represented as mean ± SEM for triplicate samples. Data were analyzed by ANOVA (analysis of variance) to compare data sets and two-sided t-tests based on the ANOVA mean squared error.

## Results

### Murine models of 5-HT infusion

At the end of 24-hour of mini pump insertion, platelets were counted in blood samples from both models, saline and 5-HT-infused mice and indicated that 5-HT infusion did not change the number of circulating platelets. The 5-HT concentrations in plasma were determined by ELISA [10], [18]. The plasma 5-HT levels were found to be 0.85±0.04 ng/µl of blood for saline-infused animals (n = 15) and 2.74±0.37 ng/µl of blood for 5-HT-infused animals (n =  15). The plasma 5-HT levels differed markedly between the two animal groups. 5-HT-infused mice showed a 3.2–fold increase in plasma 5-HT concentration.

### RNA isolation techniques

From saline- and 5-HT-treated mice, bone marrow cells were isolated from femoral bones and megakaryocytes were subsequently isolated following an established immunomagnetic technique [23], [24], [26]. A CD41^+^ monoclonal antibody, which is highly specific for mature and immature MKs, was utilized during the purification method [25], [26].

Quality control steps were carried out to ensure that high quality RNA was used for microarray and qRT-PCR analysis. Using an Agilent RNA 6000 Pico Assay and 2100 BioAnalyzer, isolated total RNA was analyzed. The RNA integrity number (RIN) was in the range of 7-9, indicating its high quality [30]. Assessment of amplification and labeling was performed by examining each sample with an Agilent RNA 6000 Nano kit. Genome-wide expression analyses of saline- and 5-HT-treated MKs were performed using MouseRef-8 v2 Expression Bead Chips and scanned with an iScan (Illumina, San Diego, CA). Data preprocessing included log transformation and median normalization. From a total of four pooled samples in each treatment group, one control sample was eliminated after determining it was an outlier.

### Gene expression analysis

We profiled the global gene expression of megakaryocyte-derived RNA from saline- and 5-HT-infused mice with the Illumina array platform using the mouse WG-6 v2.0 Expression BeadChip containing 45,281 probe sets representing 30,854 genes. The differentially expressed genes between the two groups were determined using a Bayes moderated *t*-test and implemented using the R language [28]. We have identified 5,404 genes expressed in both groups and 723 differentially expressed genes (FC>1.5 and p-value <0.05) of which 706 genes were induced and 17 genes were repressed by elevated plasma 5-HT levels.

### Differentially expressed genes

GO mining functional analysis of differentially expressed genes (FC>1.5 and p-value <0.05) was carried out by uploading the lists of genes that were increased (n = 706) and decreased (n = 17) into MetaCore software. Based on GO annotations, as shown in Table 1, most of the up-regulated genes belong to multiple functional categories that reflect a change in metabolic and molecular processes in different cellular compartments as an effect of 5-HT treatment. Among these include catabolic processes (4.7% of transcripts), cellular (3.7%) and organelle organization (4.4%), protein binding (3.2%), and catalytic activity (3.1%). Interestingly, there was a high increase of transcripts involved in oxidoreductase activity (5.1%). This possibly reflects highly active metabolic pathways especially in mitochondrial processes (5.5%) and vesicle-mediated transport (5.2%), indicating highly active trafficking between endosomes and plasma membrane. GTPase activity (6%) was also prominently increased, which is also involved in endosomal transport.

**Table 1:** Gene Ontology Mining Tool functional analysis.

GO functionalCategory	Percentage of mapped transcripts	No. of transcripts represented on the array that map to the GO category
**Up-regulated genes with 5-HT treatment**		
**Biologic Process**		
Small molecule metabolic process	4.5	2500
Catabolic process	4.7	1947
Cellular component organization	3.7	4398
Organelle organization	4.4	2090
**Cellular component**		
Cytoplasmic part	3.7	6852
Intracellular organelle	3.1	11173
Mitochondrial part	5.6	806
Vesicle-mediated transport	5.3	983
**Molecular function**		
Protein binding	3.2	8753
Catalytic activity	3.1	5980
Oxidoreductase activity	5.1	842
GTPase activity	6.0	251
**Down-regulated genes with 5-HT treatment**		
**Biologic Process**		
Nucleic acid metabolic process	0.061	4896
Cellular nitrogen compound metabolic process	0.056	5386
Cellular biosynthetic process	0.066	4525
**Cellular component**		
Cytoplasm	0.028	10700
Nucleus	0.032	6206
Intracellular organelle	0.027	11173
**Molecular function**		
Protein binding	0.023	8753

Gene list created using genes FC>1.5 and p-values≤0.05 relative to saline-infused MK pop.

On the other hand, the down-regulated genes (n = 17) were related to nucleic acid (0.06%) and cellular biosynthetic processes (0.07%), which were mainly involved in (L)-alanine, (L)-cysteine, and (L)-methionine metabolism. The inhibitory effects of 5-HT in MK cells were not as pronounced as its stimulatory effects, which can be explained by the fact that MKs harbor the 5-HT_2A_ receptor subtype like platelets [31], where it plays roles in stimulation and regulation of megakaryopoiesis [32]. As a whole, the induction of these cellular processes reflects a highly metabolic state during proplatelet formation. Consistent with what is currently known about 5-HT, it appears that its role in MKs is mainly induction of cellular and molecular process.

### Hierarchical gene clustering analysis

**Figure 1:**
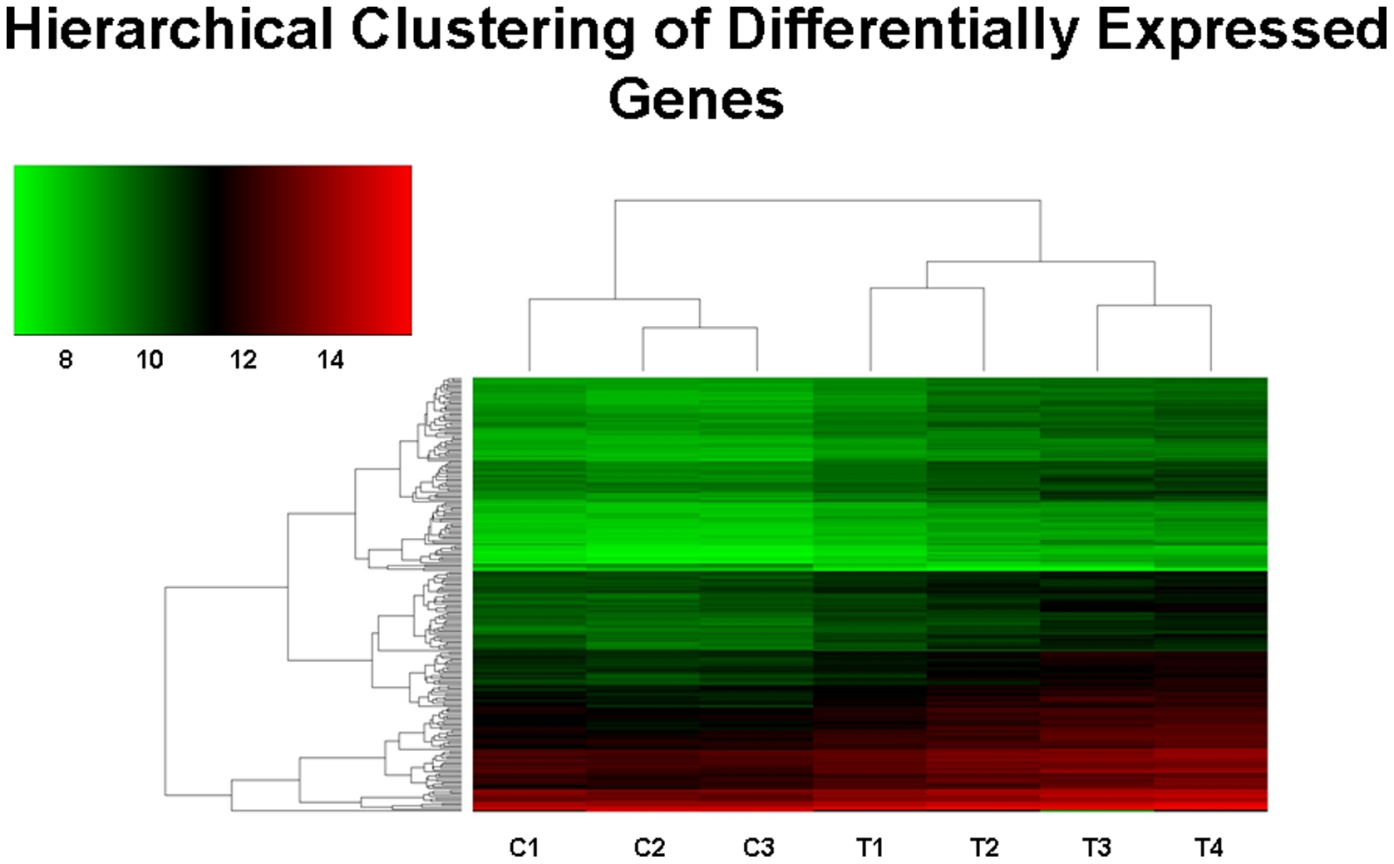
Hierarchical clustering analysis of genes differentially expressed under elevated 5-HT conditions in MK cells. The expression pattern of 233 genes whose expression was changed significantly (FC>2, p<0.001) in saline (n = 3, denoted as C1–C3) and 5-HT-treated (n = 4, denoted as T1–T4) samples are shown. The data were clustered using the standard hierarchical method with average linkage and using the Pearson correlation to determine the distance function. The normalized expression index for each gene (rows) in each sample (columns) is indicated by a color code (see Expression index bar at top left of figure). The genes shown represent the genes that were up-regulated (red) and down-regulated (green) in the sample sets. Samples with similar patterns of expression of the genes studied will cluster together, as indicated by the dendogram.

Using average linkage and Euclidean distance metrics, relations between groups and clusters were represented in a heat map. Unsupervised nearest-neighbor hierarchical clustering identified differences in the transcriptome of saline- and 5-HT-infused MK populations. Genes with FC>2 and p<0.001, which consist of 227 up-regulated and 6 down-regulated genes, were analyzed and compared between the two treatment groups (Figure 1). This revealed distinct expression patterns that highlights the stimulatory effect of 5-HT in the global gene expression of MKs.

### Functional categorization of differentially expressed genes

**Figure 2:**
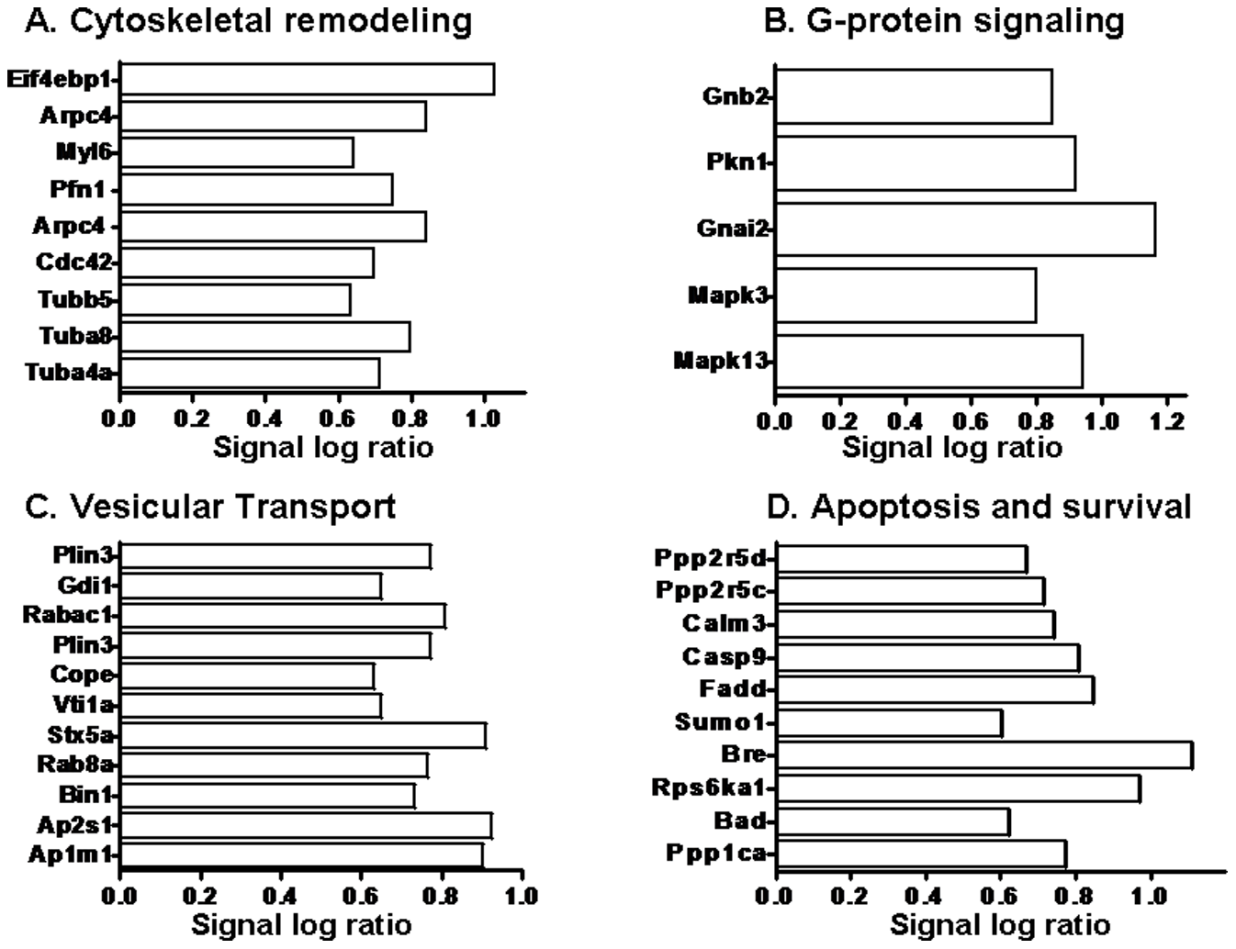
Functional classification of differentially expressed genes. Genes (FC>1.5, p<0.05) are broadly grouped into selected functional categories: (**A**) cytoskeletal remodeling, (**B**) G-protein signaling, (**C**) vesicular transport, and (**D**) apoptosis and survival. The abscissa refers to the signal log ratio (SLR), representing the log_2_ of the average change between transcript expression in saline- and 5-HT-infused megakaryocyte populations (SLR = 1 or log_2_ of 1  =  FC of 2).

To understand the biological significance of elevated plasma 5-HT levels on gene expression in MKs, differentially expressed genes were broadly grouped into functional categories. The expression levels of differentially expressed genes involved in (i) cytoskeletal remodeling, (ii) G-protein signaling, (iii) vesicular transport, and (iv) apoptosis and survival are shown in Figure 2A–D. A significant number of genes were involved in cytoskeletal remodeling which facilitates platelet shape change and cytoskeletal reorganization. The increased expression of *Myl6* and *Pfn1* induce formation of stress fibers and actin reorganization, which stimulates cell motility.

Clathrin-mediated vesicular transport is also up-regulated upon 5-HT stimulation, with increases in transcripts of *Rab8a*, *GDI1, Ap1/Ap2, Vtil1a* and *Stx5a*, which belong to the broad SNARE family of proteins. Meanwhile, apoptosis and anti-apoptosis genes are differentially expressed with 5-HT infusion, such as *Bad*, *Bre*, and *Casp9* genes which act in opposing direction, probably regulating a delicate balance between apoptosis and survival during MK maturation and platelet formation [32], [33].

### Confirmation of gene expression of specific genes

Specific genes involved in platelet biology were explored to understand the impact of elevated plasma 5-HT levels in the functional abilities of platelets. We analyzed the set of genes with FC>1.5 using MetaCore software. Table 2 lists the genes that are involved in G-protein coupled receptor (GPCR) (e.g., G-coupled 5-HT_2A_ receptor) and downstream signaling pathways that lead to activation of platelet integrins, which promote platelet communication and adhesion. The up-regulated expression of these genes is consistent with the phenotypic and functional changes demonstrated in platelets upon stimulation with 5-HT [15]–[18]. Among these are genes involved in RhoA signaling, in which the downstream effector Rho-associated coiled-coil containing protein kinase (ROCK) facilitates the phosphorylation of Cfl1 resulting in depolymerization of the actin cytoskeleton [34]. In parallel, ROCK also induces expression of *Mly6*, a myosin light chain that forms a complex with heavy myosin chains to facilitate stress fiber formation and induction of platelet shape change [35].

**Table 2:** Expression level of genes involved in GPCR-mediated platelet activation.

Official Gene Symbol	Gen Bank Accession No.	Gene Description	MicroarraySLR	
**Receptor**				
*Itga2b*	NM_010575.1	integrin, alpha 2b (platelet glycoprotein IIb of IIb/IIIa complex, antigen CD41)	1.15	
**Talin-dependent integrin activation**				
*Apbb1ip*	NM_019456	amyloid beta (A4) precursor protein-binding, family B, member 1 interacting protein	0.75	
**Enzyme**				
*Plcb2*	NM_177568.2	phospholipase C, beta 2	0.78	
**Rho Signaling**				
*Arhgef1*	NM_008488.1	Rho guanine nucleotide exchange factor (GEF) 1	0.93	
**Cytoskeletal remodeling**				
*Myl6*	NM_010860.2	myosin, light polypeptide 6, alkali, smooth muscle and non-muscle	0.65	
*Mylc2*	NM_023402.1	myosin light chain 2	0.79	
*Cfl1*	NM_007687.2	cofilin 1, non-muscle	0.78	
**Granule Secretion**				
*Stx4a*	NM_009294.2	syntaxin 4A	0.87	
*Vamp3*	NM_009498.3	vesicle-associated membrane protein 3	0.76	

**Figure 3:**
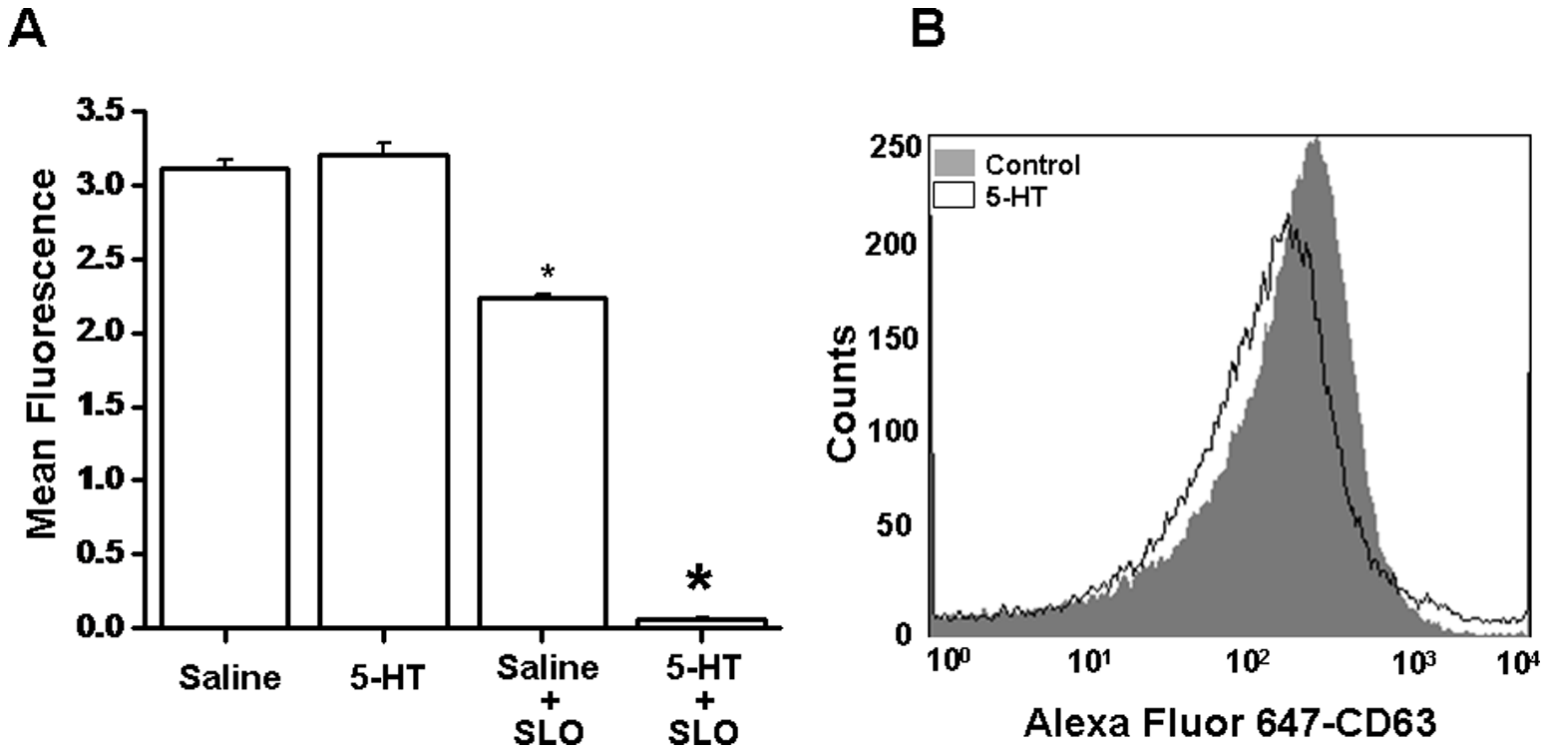
Dense-granule secretion assay. (**A**) Platelets isolated from mice after 24 hours of 5-HT infusion exhibit increased dense-granule exocytosis, as indicated by increased levels of secreted 5-HT. Dense granule secretion was monitored by measuring the level of fluorescent material which was created by OPT as described in the Materials and Method section. The measurements were done at excitation wavelength 355 nm and emission wavelength 475 nm. Data represent mean ± SD; Asterisk (*) indicates statistical significance compared to Saline+SLO by Student’s *t*-test, *p<0.05*. (**B**) Dense-granule secretion was monitored following plasma membrane staining for granulophysin (CD63), a surrogate marker of exocytosis. FACS analysis demonstrates increased mean fluorescence intensity following 5-HT infusion compared to saline-infused littermates.

Consistent with what is known in the literature, SNARE proteins, which facilitate exocytosis of platelet granules [36], [37], are also up-regulated as a function of elevation in serum 5-HT levels. Our data showed an elevation in the level of *Stx4a* and *Vamp3* transcripts. Stx4a binds to SNAP25, Rab4, and Vamp3 proteins, which are suggested to be involved in the turnover of platelet alpha- and dense-core granule exocytosis [15]–[18]. In correlating the elevated expression of MK genes in platelets, the dense granules secretion rates were compared between platelets of saline- and 5-HT-infused mice. We adopted and modified a previously described method for measuring 5-HT release using streptolysin-O permeabilized platelets [29]. It is well established that ATP is essential for the secretion of dense granules, whereby in the absence of ATP, Ca^2+^ could not induce 5-HT secretion [16], [29]. The Ca^2+^-induced secretion of dense granules was efficiently reconstituted by the addition of ATP in the secretion buffer. On incubation of permeabilized platelets, Ca^2+^-induced dense-granule secretion of 5-HT was significantly altered between the mice groups (Figure 3A). Upon platelet permeabilization, secretion of 5-HT was induced by 90% in 5-HT-infused mice compared to their control littermates. This coincides with the increased plasma membrane expression of the surrogate marker granulophysin (CD63), which is a marker of dense-granule exocytosis (Figure 3B).

**Figure 4:**
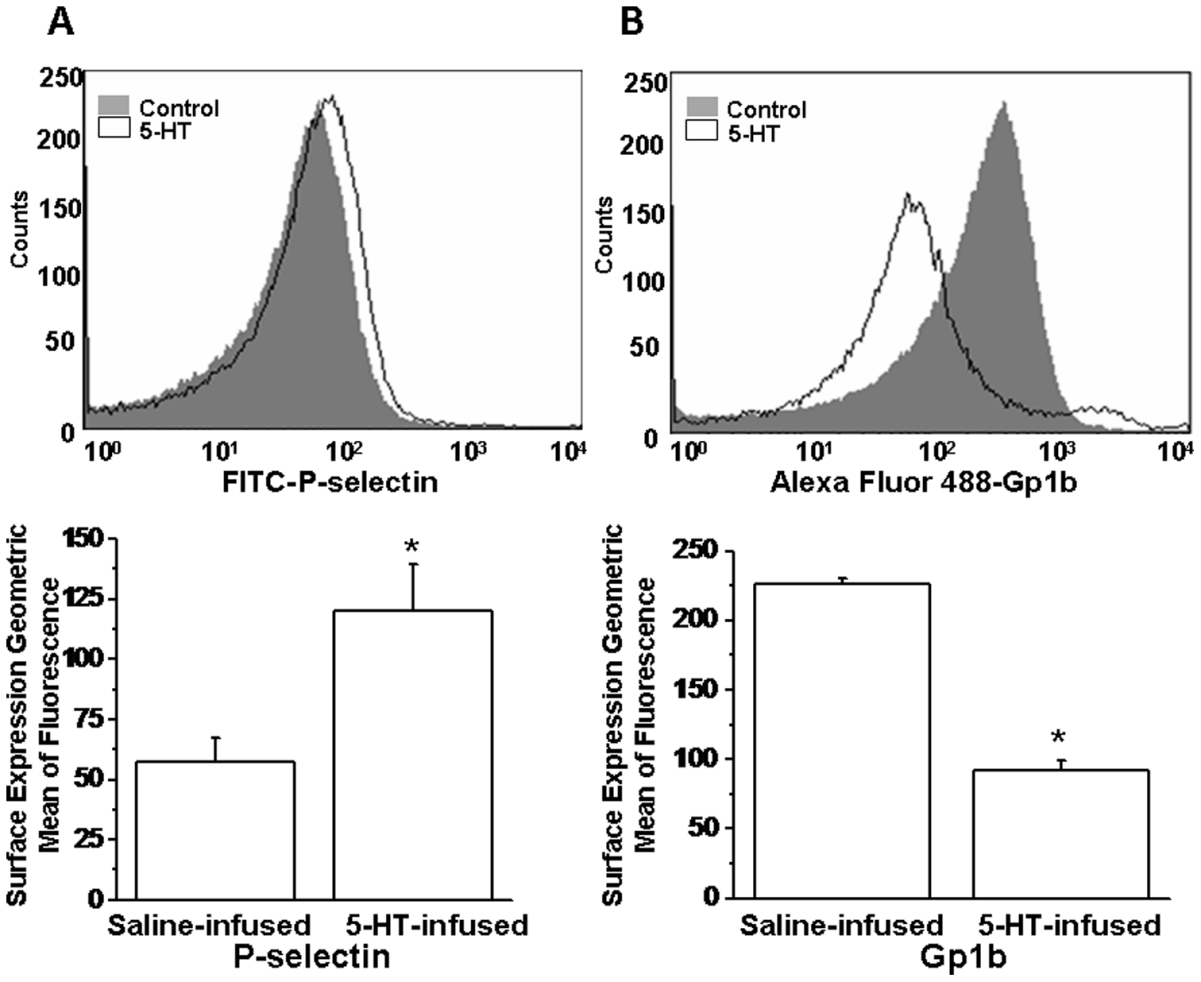
Cell-surface expression of surrogate markers of platelet activation. (**A**) FITC-labeled P-selectin (CD62P) and (**B**) Alexa Fluor 448-labeled Gp1b show increased and decreased mean fluorescence intensity (unshaded area), respectively, following *in vivo* 5-HT treatment in contrast to saline-infused littermates (gray shaded area); n = 5–6. Increased staining indicates active exocytosis of alpha-granules in platelet membranes whereas decreased expression of Gp1b is consistent with enhanced platelet-platelet binding in the presence of high 5-HT. The assay was performed in triplicate.

The elevation in membrane trafficking also establishes the activation of inside-out signaling, brought about by an increase in free intracellular Ca^2+^ stores and diacylglycerol (DAG), which occur primarily through protein kinase C (PKC) [38]. In an alternative pathway, integrin activation of the complex alpha-IIb/beta-3 is accomplished via the CalDAG-GEF/RasGRP protein family (calcium-binding EF hands and DAG-binding C1 domains) and the small GTPase Rap1 [35], [38]–[40], sending outward signals for platelet aggregation and adhesion. Although in 5-HT-infused MKs, the expressions of *Selp* (P-selectin) and *Gp1b* (glycoprotein 1b) genes were not altered, their protein levels on platelet plasma membranes were found to be different than platelets obtained from saline-infused mice (Figure 4). These findings are in good agreement with the 5-HT-mediated elevation of the MK genes proposed to be involved in membrane trafficking in platelets, such as PKC-phosphorylation of P-selectin, which moves it to the platelet plasma membrane [41].

## Discussion

Numerous studies have characterized the roles of 5-HT at elevated plasma levels in different tissues. However, to the best of our knowledge the impact of this multifunctional compound on gene expression, more specifically in MK cells, has not been identified prior to this study. Here, MK-derived mRNA isolated from 5-HT-infused mice was analyzed using a microarray platform and compared with MK mRNA prepared from saline-infused mice. MKs, which are direct precursors of platelets, undergo a series of intricate processes and replication forming giant cells with a polyploid state (up to N = 128) [31]. Similar to platelets, MKs express the 5-HT_2A_ receptor subtype [32], which makes them susceptible to the effects of circulating plasma 5-HT. Bioinformatics tools were employed to determine the genes and pathways that were regulated in mice following 5-HT infusion. The up-regulated genes identified were broadly classified into categories which include cytoskeletal remodeling (*Myl6, Pfn1, Cec42, Tuba8*), G-protein signaling (*Gnai2, Map3k, Map13k*), membrane trafficking (Rab8a, Stx4a, *Vamp3*), and apoptosis and survival (Fad, Bad, Casp9). The activation of apoptosis in MKs is a hallmark of the terminal stages of MK development [31], in which the fragmentation of MK progenitor cells marks the formation of platelets and its subsequent release in the peripheral circulation. *In vitro* studies have shown that the activation of the apoptotic cell machinery terminates MK development and initiates the formation of millions of platelets [31]–[33]. Although there are different pathways activated that lead to apoptosis, in our model, it appears that the adaptor protein Fadd (Fas-associated death domain protein) is synthesized and the Fas-pathway is activated. Following this activation, *Bcl* pro-apoptotic genes, including *Bad*, *Bax*, and *Bnip3,* are transcribed, which are involved in the regulation of the mitochondrial membrane permeability complex during apoptosis [31]–[33]. Downstream to this signaling cascade is the recruitment of cysteine-dependent aspartate-specific proteases, like caspase 9, which terminates in the proteolytic cleavage of proteins [33]. This process, described as both intricate and unusual compared to other hematopoietic cell lines, terminates the MK life cycle. Along with the activation of apoptosis, other critical steps in proplatelet formation are cytoskeletal remodeling and microtubule extension, allowing movement of cargo proteins, formation of new vesicles, and incorporation of newly synthesized proteins into new organelles.

Earlier, we showed that in 5-HT-infused mice compared to saline-infused littermates, the movement of cytosolic proteins to the plasma membrane is changed [17], [18] as evidenced by increased plasma membrane staining for granulophysin (CD63) and P-selectin (CD62), which are the surrogate markers of alpha- and dense-granules (Figures 3B and 4A). These markers indicate constitutive exocytosis of the procoagulant vesicles, probably until these platelets are recruited into a growing thrombus *in vivo* [18]. Therefore, verifying our study model using 5-HT-infused mice, changes in plasma 5-HT level was correlated with phenotypic changes observed in platelets as a function of 5-HT infusion (Figure 3). In contrast, Gp1b (CD42) which is a component of the Gp1b-V-IX complex, is the initial site for vWF factor that allows aggregatory responses in platelets. Upon activation, plasma membrane expression is decreased (Figure 4B), most likely as a result of the internal redistribution of the receptor.

Platelet dense-granules are specialized secretory organelles that store high concentrations of 5-HT, along with small procoagulant molecules such as ADP, ATP, and Ca^2+^ that can be mobilized during platelet activation. Small GTPases, like Rab4, undergo serotonylation [15]–[18], which renders them into a constitutively active GTP-bound form and facilitates the exocytosis of 5-HT-rich dense-granules. Following 5-HT stimulation, 5-HT stores are mobilized and secreted in permeabilized platelets, which in effect amplifies the external 5-HT signal by increasing the extracellular concentration of 5-HT. Previous studies identified certain proteins that modulate the trafficking and docking of secretory granules in using GTPases [15]–[18], [42] and SNARE proteins [36].

The binding of 5-HT to the 5-HT_2a_ receptor initiates a signal transduction that culminates in platelet shape change, promotion of granule secretion, and the activation of integrin GPIIb/IIIa [15]–[18]. The activation of the RhoA/ROCK pathway [34] allows cytoskeletal remodeling and formation of platelet extensions, affecting platelet shape change from discoid into a stellate conformation, via myosin II and actin [35]. However, in a receptor-independent pathway, 5-HT in the platelet cytosol is transamidated on small GTPases converting them to their active, GTP-bound form [15]–[18], [42], [43]. One of the downstream events is an association between Rab4-GTP is the movement of the granules to the plasma membrane, more specifically exocytosis of alpha-granules [15], [16], [42], [43]. These findings verified the impact of elevated plasma 5-HT level on platelets and confirmed the changes in expressions of MK genes due to the elevated 5-HT.

Additional studies are necessary to elucidate the signaling mechanisms by which the MK located 5-HT_2A_ receptor responds with elevations in plasma 5-HT and to delineate the role of changed gene expressions in platelets. In this regard, our novel findings serve to provide initial evidence that increased plasma 5-HT is linked to a dramatic global change in MK gene expressions potentially leading to altered interaction of platelets with other platelets or with the blood vessel wall.
